# Weight Variation over Time and Its Association with Tuberculosis Treatment Outcome: A Longitudinal Analysis

**DOI:** 10.1371/journal.pone.0018474

**Published:** 2011-04-08

**Authors:** Antonio Bernabe-Ortiz, Cesar P. Carcamo, Juan F. Sanchez, Julia Rios

**Affiliations:** 1 Epidemiology Unit, School of Public Health and Administration, Universidad Peruana Cayetano Heredia, Lima, Peru; 2 CRONICAS, Center of Excellence in Chronic Diseases, Universidad Peruana Cayetano Heredia, Lima, Peru; 3 Parasitology Department, US Naval Medical Research Unit No. 6 (NAMRU-6), Lima, Peru; 4 National Health Strategy for Control and Prevention of Tuberculosis, DISA II Coordinator, Lima, Peru; San Francisco General Hospital, University of California San Francisco, United States of America

## Abstract

**Objective:**

Weight variation during therapy has been described as a useful marker to predict TB treatment outcome. No previous study has used longitudinal analysis to corroborate this finding. The goal of this study was to evaluate change and trends of patients' bodyweight over time depending on TB treatment outcome.

**Methods and Findings:**

A retrospective cohort study with all TB cases diagnosed from 2000 to 2006 was carried out. Information from 5 public tuberculosis treatment facilities at Pampas de San Juan de Miraflores, Lima, Peru was analyzed. Poor outcome was defined as failure or death during TB therapy, and compared to good outcome defined as cured. Longitudinal analysis with a pre-specified marginal model was fitted using Generalized Estimating Equations to compare weight trends for patients with good and poor outcome adjusting for potential confounders. A total of 460 patients (55.4% males, mean age: 31.6 years) were included in the analysis: 42 (9.1%) had a poor outcome (17 failed and 25 died). Weight at baseline was not different comparing outcome groups (p = 0.17). After adjusting for age, gender, type of TB, scheme of treatment, HIV status and sputum variation during follow-up, after the first month of treatment, patients with good outcome gained, on average, almost 1 kg compared to their baseline weight (p<0.001), whereas those with poor outcome lost 1 kg (p = 0.003). Similarly, after 4 months, a patient with good outcome increased 3 kg on average (p<0.001), while those with poor outcome only gained 0.2 kg (p = 0.02).

**Conclusions:**

Weight variation during tuberculosis therapy follow-up can predict treatment outcome. Patients losing weight during TB treatment, especially in the first month, should be more closely followed as they are at risk of failure or death.

## Introduction

During 2009, 9.4 million new tuberculosis (TB) cases were estimated with about 3% occurring in the Americas [1]. An estimated 440,000 cases were caused by multi-drug-resistant TB (MDR-TB) and Peru is one of the countries reporting cases of extensively drug-resistant TB (XDR-TB) [1].

In most countries, TB patients usually receive MDR-TB testing if they fail to treatment after surviving at least 5 months of empiric therapy with a standardized first-line antibiotic regimen [2], [3]. Peru has the highest rate of MDR-TB in the Americas, with 5.3% MDRTB in new TB cases, 24% in re-treatment cases and more than 200 cases of XDR-TB reported by 2008 [1], [4].

TB is a wasting disease [5], [6], [7], [8] and bodyweight variation has been proposed as a practical anthropometric marker to predict TB treatment outcome [9], [10], [11], [12]. Moreover, weight loss of 2 kg. or more during the first-month therapy has been considered as a potential risk factor for toxicity due to drugs [13]. Many countries, including Peru, routinely weigh patients and repeat sputum microscopy tests on a monthly basis during therapy to assess treatment response. Several studies have reported that positive sputum microscopy at second month of treatment is associated with subsequent treatment failure, but is insensitive at population level [2], [14], [15], [16]. Thus, patients' bodyweight might be a helpful and cheap test to predict TB treatment outcome. Although many papers have reported bodyweight as a marker to predict therapy failure, death or relapse, to our knowledge, no study has reported an appropriate longitudinal analysis of patients during TB treatment assessing bodyweight change over time. Longitudinal data analysis is a statistical technique that allows the direct study of within-individual change over time accounting for within-individual correlation in the analysis [17].

The objective of this paper was to assess change of patients' bodyweight over time depending on TB treatment outcome. This model was adjusted for several potential confounders. We hypothesized that the trends of patients' bodyweight with poor outcome, those who had died or failed during treatment, differed from those who had ended treatment as cured.

## Materials and Methods

### Study design, setting and participants

Data from a retrospective cohort of patients commencing tuberculosis therapy was used and analyzed for this study. This research used programmatic TB diagnosis, treatment monitoring and outcome data as recorded from the National Health Strategy for Prevention and Control of Tuberculosis (ESN-PCT) at 5 public tuberculosis treatment facilities in Pampas de San Juan de Miraflores, a periurban shantytown located at the south of Lima, Peru.

Patients included in the analysis were at least 18 years old and diagnosed with tuberculosis disease between 2000 and 2006. We deliberately choose these years because changes on TB treatment monitoring occurred starting 2007 in the ESN-PCT. We excluded patients if they were restarting partially completed or interrupted TB treatment or therapy had failed within the previous 12 months because this group has a high level of drug resistance and therefore, evolution of these patients is completely different [2], [3].

### Outcomes and variables of interest

The main outcome of the study was bodyweight, recorded in kilograms (kg) from treatment start (baseline) and repeatedly measured in a monthly basis. For this study, only data from the first 5 months were used for analysis.

The ESN-PCT defines treatment failure based on positive sputum microscopy results after ≥5 months of treatment or reverting to positive after two consecutive negative monthly results [18]. Deaths were defined as those patients who died during tuberculosis therapy follow-up. For our research, we dichotomized these programmatic outcomes in ‘cured’ (good outcome), or to have had an adverse or poor outcome (death or treatment failure, as previously defined).

Other variables of interest included in the analysis were: age measured in years, patient sex (male or female), type of tuberculosis (pulmonary vs. extra-pulmonary), treatment scheme (new vs. recurrent), change of sputum during treatment assessed monthly, and HIV status (positive vs. unknown). Since HIV test is not habitually performed among TB cases and no patients was found to be HIV negative, we categorized this variable as positive or unknown.

### Procedures

All patients were treated by the TB programme with empiric standardized first-line therapy and clinic-based direct-observation of every dose (DOTS approach) [19]. The Peruvian TB programme uses monthly food packages as adherence incentives and these are standardized and given for all patients. Management of TB cases included programmatic monitoring with monthly sputum microscopy and weight (kg) measures [18]. For microscopy, the TB programme used un-concentrated direct Ziehl-Neelsen smear microscopy with a well-established national quality assurance system. For weighing, data from records taking from the existing clinic scales and established programmatic training for their use was utilized. The accuracy of the scales used was not systematically confirmed but each patient was weighed repeatedly using the same scale and weights were generally recorded to the nearest 0.1 kg.

### Data analysis

Data were analyzed using STATA version 11.0 for Windows (STATA Corporation, College Station, Texas, US). First, a brief description of demographic and clinical characteristics was tabulated. Second, weight was calculated for each group according to our outcome of interest and month of follow-up. Finally, a longitudinal analysis was carried out to evaluate weight change over time. A marginal model was fitted using Generalized Estimating Equations to model average weight trends for patients with good and poor outcome [20]. The crude model was specified as follow:
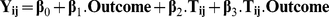
Where Y_ij_ is the mean weight (kg) in patient “i” at time “j”, β_0_ is the intercept, i.e. weight in kilograms among those with good outcome at baseline, β_1_ is the difference in weight in patients with poor outcome compared to those with good outcome at baseline, β_2_ quantifies the change in weight between baseline and one selected month for participants with good outcome, and the sum of β_2_ and β_3_ (interaction term) represents the change in weight between baseline and one selected month for participants with a poor outcome [17]. In this model, the time variable was included as categorical because weight over time did not show linearity in the poor outcome group.

Quasi-likelihood under the independence model information criterion (QIC) [20], an extension of the Akaike's information criterion (AIC), was applied to find the best working correlation structure applicable for the proposed model. Additionally, the model was adjusted for potential confounders affecting both outcome and weight. Potential confounders included were age, sex, type of tuberculosis, treatment scheme, HIV status, and sputum microscopy result change during treatment. Wald test was used to report p-values, whereas robust standard errors were used to calculate 95% confidence intervals for each coefficient in the model.

### Ethics issues

Institutional review board (IRB) approval for this project was granted by Universidad Peruana Cayetano Heredia. Informed consent was waived by IRB because of use of routine and programmatic data of the National Health Strategy for Control and Prevention of Tuberculosis.

## Results

### Description at baseline

A total of 530 patients started tuberculosis treatment during the period of study and were eligible for this study; but, 20 moved away before starting treatment, 37 abandoned therapy, 11 had previous failures, and 2 had unknown outcomes. Therefore, 460 (87%) patients were included in the analysis, 55.4% of them were males and the mean age was 31.6 years (SD: 14.1; range: 18–80). Of the total, 42 (9.1%) had a poor outcome at the end of tuberculosis therapy (17 failed and 25 died). A brief description of patients' characteristics in relation to outcome status is shown in Table 1.

**Table 1:** Characteristics of enrolled patients at baseline according to outcome status*.

Variable	Good outcome(n = 418)	Poor outcome(n = 42)	p-value
***Sex***			
Female	187 (44.7%)	18 (42.9%)	0.82
Male	231 (55.3%)	24 (57.1%)	
***Age (years)***			
Mean (SD)	30.8 (13.2)	39.4 (19.5)	<0.001
***Type of tuberculosis***			
Extra-pulmonary	67 (16.1%)	7 (16.7%)	0.92
Pulmonary	350 (83.9%)	35 (83.3%)	
***Scheme of treatment***			
New	360 (86.1%)	28 (66.7%)	0.001
Recurrent	58 (13.9%)	14 (33.3%)	
***Sputum result***			
Negative	118 (29.0%)	9 (23.1%)	0.64
1+	155 (38.1%)	15 (38.5%)	
2+	73 (17.9%)	10 (25.6%)	
3+	61 (15.0%)	5 (12.8%)	
***HIV infection*** **			
Unknown	414 (9.0%)	39 (92.9%)	0.002
Yes	4 (1.0%)	3 (7.1%)	
***Days of follow-up***			
Mean (SD)	196 (38.6)	134 (74.9)	<0.001

*Results may not add due to missing values.

**Only 7 patients were known to be HIV-positive because HIV testing is rarely performed.

### Weight during treatment follow-up

Table 2 shows a detailed description of weight variation during treatment follow-up without accounting for intra-subject correlation. There was no significant difference between weights of outcome groups at baseline (p = 0.12); however, on average, weight decreased in those who developed an adverse outcome whereas it increased among those who ended treatment as cured.

**Table 2:** Weight change over time during follow-up according to outcome status.

Weight (kg)	Treatment outcome			
	Good outcome		Poor outcome	
	N	Mean (SD)	N	Mean (SD)
Baseline	418	54.7 (8.3)	42	52.5 (9.2)
First month	412	56.0 (8.4)	39	50.6 (9.7)
Second month	405	56.8 (8.5)	33	49.5 (10.3)
Third month	401	57.7 (8.3)	29	51.7 (10.2)
Fourth month	389	58.3 (8.4)	26	53.7 (8.7)
Fifth month	389	58.7 (8.7)	18	51.0 (13.1)

### Weight change over time

When assessing correlation structure for repeated measurements using QIC, the best working correlation was exchangeable. Other structures (auto-regressive, unstructured, and non-stationary) were evaluated with the model, but they did not achieve convergence. In any case, robust standard errors were used to handle misspecification of variance or correlation functions [20].

**Figure 1:**
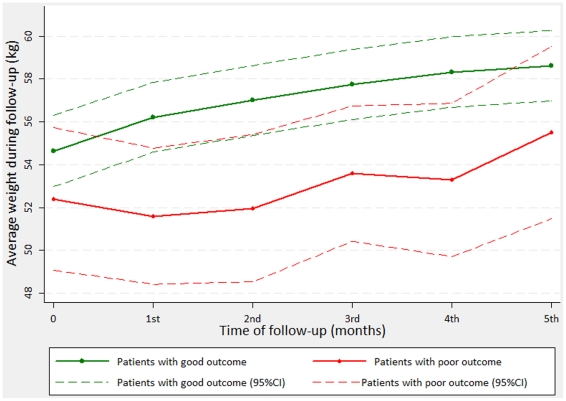
Weight change over time during treatment follow-up according to outcome status (adjusted model*). * Predicted lines were adjusted for age, gender, type of tuberculosis, scheme of treatment, HIV status, and sputum variation during follow-up.

Results of crude and adjusted marginal models are shown in Table 3. Of interest, adjusted coefficient for adverse outcome was not significant (p = 0.17), indicating that the difference in weight (about 2 kg) among patients with poor and good outcome at baseline was not statistically different. However, the interaction terms together were significant (Wald test for interaction, p = 0.002) indicating that changes of weight over time among patients with poor outcome differed of those with good outcome (Figure 1). Based on the results of the adjusted model (Table 3), at the end of the first month, on average, patients with good outcome gained almost 1 kg (0.93 kg according to the adjusted model) compared to their baseline, whereas at the fourth month, weight increased about 3 kg. On the other hand, patients with poor outcome lost about 1 kg (0.97 kg according to the model) at the first month of therapy compared to the baseline, while gaining 0.2 kg after four months of treatment. Moreover, patients with poor outcome did not gain weight during the first two months of therapy.

**Table 3:** Crude and adjusted marginal models assessing weight change over time according to outcome status.

	Crude model			Adjusted model*		
	β	95%CI	p-value	β	95%CI	p-value
Intercept	54.70	53.90; 55.50	<0.001	56.91	53.09; 60.73	<0.001
Poor outcome	−2.25	−5.13; 0.64	0.127	−2.07	−5.04; 0.90	0.172
Time (1st month)	1.46	1.24; 1.68	<0.001	0.93	0.54; 1.31	<0.001
Time (2nd month)	2.24	1.96; 2.52	<0.001	1.67	1.24; 2.10	<0.001
Time (3rd month)	3.01	2.68; 3.33	<0.001	2.42	1.95; 2.89	<0.001
Time (4th month)	3.58	3.23; 3.92	<0.001	2.97	2.49; 3.46	<0.001
Time (5th month)	3.93	3.56; 4.30	<0.001	3.33	2.82; 3.84	<0.001
Poor outcome* Time (1st month)	−2.35	−3.54; −1.15	<0.001	−1.90	−3.16; −0.64	0.003
Poor outcome* Time (2nd month)	−3.18	−4.98; −1.39	0.001	−2.56	−4.32; −0.80	0.004
Poor outcome* Time (3rd month)	−2.90	−5.16; −0.64	0.012	−2.05	−3.64; −0.46	0.011
Poor outcome* Time (4th month)	−3.41	−5.58; −1.25	0.002	−2.81	−5.15; −0.48	0.018
Poor outcome* Time (5th month)	−3.07	−5.75; −0.39	0.025	−1.25	−3.76; 1.27	0.331

*Adjusted by age, gender, type of tuberculosis, scheme of treatment, HIV status, and sputum variation during follow-up.

## Discussion

This study shows that, after adjusting for potential confounders, the curve of weight over time among patients who developed adverse outcome is completely different from patients classified as cured at the end of follow-up. The association continues being statistically significant after having included the monthly sputum microscopy result as confounder [14], [15], [21], [22], pointing out that change in weight over time is an independent predictor of treatment outcome.

These findings might have an important impact on public health, especially in resource-constrained settings. Weight assessment might be an easy, cheap, and useful form to predict TB treatment outcome among patients receiving therapy. Weight after the end of the first month of therapy, characterized by bodyweight loss among those with poor outcome, might be very important to avoid deaths or failures. Remarkably, most of the divergence of weight over time occurred during the first month. After that, weight gain among poor outcome patients shows parallel trends compared to good outcome ones with, however, a lower rate. Prospective studies with standardized measurements are needed to corroborate these findings.

Tuberculosis is the archetypal wasting disease. The association of TB and nutrition status has long been evident, as older terms were used for tuberculosis such as the Greek term “phthisis” or “to waste away” [23]. Some current guidelines mention that weight loss may indicate incipient treatment failure and have been recently included in our National Health Strategy for Control and Prevention of Tuberculosis [18]. Recently, some studies have started to report that weight loss should be considered as clinically relevant [9], [10], [11], [12], [24]. One previous study reported that moderate and severe malnutrition was a risk factor associated with early death during TB treatment in rural areas of Malawi [25]. However, to our knowledge, no study has used longitudinal data analysis to show changes and trends of patients' bodyweight during treatment follow-up.

Previous studies have suggested an association between meager weight gain during tuberculosis therapy and risk of poor treatment outcome [11], [12] or relapse [10]. One of these studies reported that patients under DOTS gained 3.2 kg on average at the end of treatment. We found similar results in our good outcome group (3.3 kg at the end of five months of therapy) [12]. The other two studies identified a cutoff of 5% weight gain to predict tuberculosis treatment outcome [10], [11]. Khan et al used the 5% cutoff at the end of the intensive therapy phase (first two months) [10], whereas Krapp et al reported the usefulness of the same cutoff but at the end of the therapy [11]. Our findings suggest that we can apply strategies as soon as the end of the first month to avoid deaths and failures, including MDR testing, supplemental nutrition and closer monitoring. A total of 16 of 17 patients who failed treatment in this study were diagnosed as MDR-TB cases after failing (data not shown). On the other hand, other two different studies have shown that appropriate therapy is associated with progressive nutritional recovery and restoration of nutrition-related markers [7], [26], but this cannot guarantee appropriate body restitution measured as total arm muscle circumference, fat mass, serum albumin, bone minerals and protein mass, despite marked weight gain in patients.

Strengths of this study include the use of programmatic data to assess bodyweight change among TB patients commencing treatment; the use of longitudinal analysis with the best working correlation structure taking into account several potential confounders including changes on sputum microscopy results during follow-up, one of the well-known predictors of TB treatment outcome; and the assessment of weight trends during 5 consecutive months after beginning therapy. Many studies have reported findings using bodyweight variation during the first two months [10], [13], or at the end of therapy [11], [12], [27], [28], but not analyzed trends of bodyweight over time.

However, this study has also several limitations. First, we joined potential treatment outcomes which might lead to misclassification. Although, we collected 6-years information, we only found a small number of failures and deaths occurred during the period of the study. However, our findings agreed with previous reports showing the association between weight and TB treatment outcome. Further studies with greater sample sizes are needed to corroborate our findings. Second, socioeconomic information, a very important variable to predict patients' weight, was not available from data. Nevertheless, our cohort was located in a poor, periurban shantytown community where members of TB-affected families have been determined to live on less that US$ 1 dollar per day. Third, during the period of the study, HIV infection status was not routinely determined among TB patients. As a result, misclassification can have occurred because cases detected were diagnosed due to suspect for the presence of symptoms or opportunistic infections. HIV prevalence found in this study (1.5%) was similar to previous reports in Peru though [29], [30]. Finally, deaths due to TB occurred during the first months of therapy (64% of deaths took place before completing four month of therapy, which might have reduced the power to detect difference of weights at the fifth month of follow-up (Table 3). However, the use of generalized estimating equations can protect against missing data if this is believed to be at random. In addition, the idea of the paper was to evaluate how trends of bodyweight change would have occurred in real life using programmatic data for this purpose.

In summary, our findings reveal that trends and change of weight during tuberculosis therapy can predict treatment outcome. Thus, weight loss during the first month of therapy should be used as part of routinely clinical evaluation to take appropriate decisions. These patients should be more closely followed as they are at risk of adverse outcomes. Follow-up monitoring might include MDR diagnosis testing, supplemental nutrition, closer monitoring, HIV infection status, treatment of opportunistic infections, etc. Further studies are needed to find appropriate weight loss cutoff including sensitivity and specificity analysis, and assess the combination of sputum microscopy results with weight change over time to predict TB treatment outcomes.
